# Diet Modifications in Primary Prevention of Asthma. Where Do We Stand?

**DOI:** 10.3390/nu13010173

**Published:** 2021-01-08

**Authors:** Barbara Sozańska, Hanna Sikorska-Szaflik

**Affiliations:** Department and Clinic of Paediatrics, Allergology and Cardiology Wrocław Medical University, ul. Chałubińskiego 2a, 50-368 Wrocław, Poland; hannasikorska@gmail.com

**Keywords:** diet, asthma, allergy, primary prevention

## Abstract

The steep increase in asthma prevalence, observed worldwide in recent decades, has created an urgent need to search for effective methods of its prevention. Among other environmental factors, changes in diet habits and the potential influence of individual food components on immunological processes have been extensively studied as a potential method of intervention in primary prevention of asthma. The preventive role of some nutrients has been confirmed: unpasteurized milk reduced the risk of asthma in epidemiological studies, vitamin D supplementation was effective in preventing the transient forms of wheezing in small children and high maternal intake of fish oil reduced the risk of persistent wheeze and asthma in children. However, not all studies provided consistent results, and many food ingredients are still pending for defining their role in asthma development. Moreover, a novel approach looking not only at single food ingredients, but the whole dietary patterns and diversity has recently been proposed. In this paper, we discuss the current role of nutrients in asthma primary prevention and the reasons for inconsistencies in the study results. We look at single diet components, but also the whole dietary patterns. We describe the proposed mechanisms of action at different stages of life, identify the role of modifiers and delineate future perspectives on the application of nutrients in targeting strategies for asthma primary prevention.

## 1. Introduction

Asthma is a complex disease with a genetic and environmental background. Its definition, mainly based on symptoms description, does not differentiate various underlying pathologies. The heterogeneity of asthma pathogenesis makes the search for primary prevention strategies difficult. To date, effective methods are very limited. They are focused on avoiding the environmental risk factors (cigarette smoking, air pollution, molds, dampness or obesity) or on identifying protective factors (farm living, diverse environmental microbiota exposure). Most of these factors exert their influence mainly in early childhood or even in pregnancy. In the light of a steep increase in asthma prevalence, primarily in high-income countries and, nowadays, also in low- and middle-income areas, there is an urgent need to find new methods of primary prevention [1].

Nutrients can exert a variety of effects in allergic diseases and asthma. They modulate a developing immune system, shape the quality and diversity of the human microbiome and influence gene expression through epigenetic interactions. Food may be an allergen exacerbating the course of asthma but may also serve as a protective factor. Drinking unpasteurized milk reduced the risk of asthma, vitamin D supplementation was effective in preventing the transient forms of wheezing in small children and high maternal intake of fish oil reduced the risk of persistent wheeze and asthma in children [2,3,4]. These and other recent findings regarding the role of nutrients in the prevention of asthma opened a new prospect of possibilities. Moreover, a novel approach looking not only at a single food ingredient but the whole diet patterns and diet diversity has recently been proposed [5]. Despite the complexity of variables (food exposure period and time, doses, frequency, mutual interactions, etc.), mechanisms and outcomes (different asthma endotypes and phenotypes), applying the diet as a method of primary prevention of asthma seems to be a tempting prospect.

**Figure 1:**
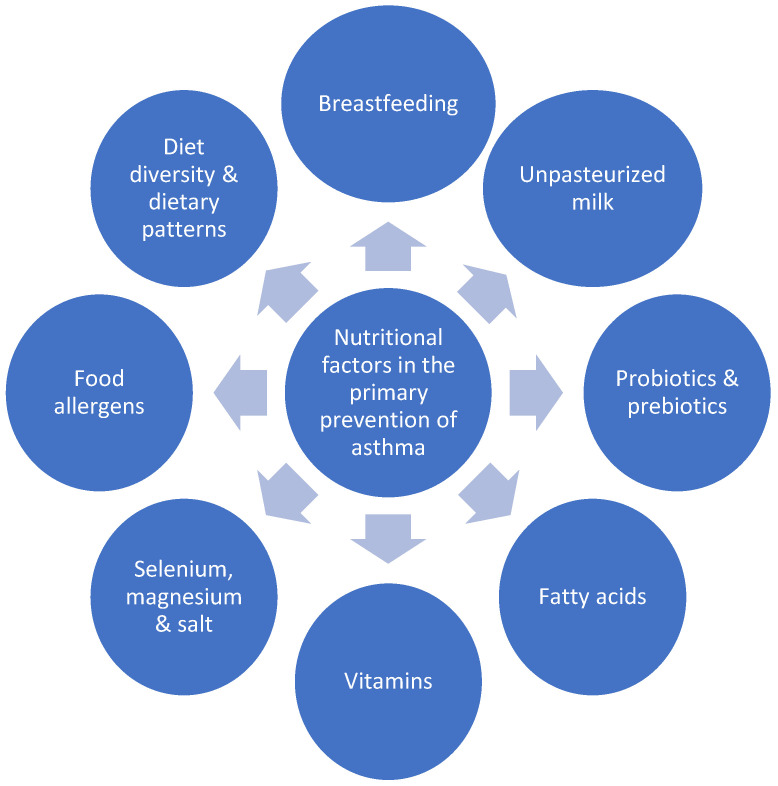
Nutritional factors with possible impact on the primary prevention of asthma.

In this paper, we aim to discuss the current knowledge about nutrients application in asthma primary prevention (Figure 1). We present the newest research, both on single components of the diet and the whole dietary patterns, propose mechanisms of action at different stages of life and discuss future perspectives.

## 2. Breastfeeding and Infant Milk Formulas

**Figure 2:**
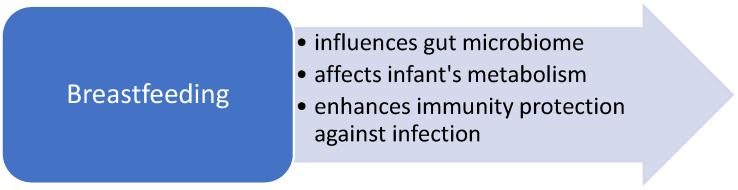
Proposed mechanism of action of breastfeeding in asthma primary prevention.

Human breastmilk is an optimal nutritional supply for neonates and infants. It influences the gut microbiome, infant’s metabolism and immunity protection against infection (Figure 2). There is strong evidence based on a large number of studies of a reduction in severe respiratory infections in breastfed children [6]. However, its role in asthma prevention is less documented and the evidence is inconclusive.

In the earliest meta-analysis, it was shown that exclusive breastfeeding for at least 3 months may reduce the risk of asthma in the first years of life by 30%. This effect was stronger in atopic families than in children with no family history of atopy [7]. These observations were not confirmed in another meta-analysis, including more subjects, where no effect of breastfeeding for 3 or 4 months on asthma and current wheeze was seen in children older than 5 years [8]. However, in more recent studies, breastfeeding longer than 6 months reduced the risk of asthma and wheeze in younger children (up to 2 years old) [9] and in older than 5 years ever breastfed, but this last association became non-significant when only cohort studies were taken into consideration [10].

The duration of exclusive breastfeeding and time of solid foods introduction may influence the development of allergies [11]. In the Influences of Lifestyle-Related Factors on the Immune System and the Development of Allergies in Childhood study (LISA), delayed introduction of solid foods (after 4 or 6 months) in infants was not protective on the development of asthma at 6 years of age [12]. Moreover, in another prospective cohort study incorporating solid foods before six months of age, this decreased asthma risk at age 5. The total duration of breastfeeding in this population influenced asthma prevention more significantly than the time of exclusive breastfeeding [13]. 

If breastfeeding is not possible, alternatively, infant milk formula or cow’s milk can be introduced to the diet. The association between feeding the baby with milk other than breastmilk in the first six months of life and the development of persistent asthma in 3-year-old children was examined. In this population, early introduction of infant formulas increased the risk of asthma almost 2-fold [14], suggesting the protective role of exclusive breastfeeding in asthma. American Academy of Pediatrics guidelines propose that in infants with a high risk of atopic diseases who cannot be exclusively breastfed, the use of hydrolyzed formula in place of a standard cows’ milk formula can help in prevention of atopic diseases [15]. However, the results from the prospective, randomized, double-blind German Infant Nutritional Intervention (GINI) study did not confirm this. Comparing the effect of hydrolyzed infant formulas with standard ones as the only substitute to breastfeeding in the first four months of life, it was shown that the risk of early asthma cannot be decreased by such intervention in infants at high risk of allergic diseases [16].

Despite the controversies and not fully documented role of mother’s milk in asthma prevention, it should be the first-choice type of feeding recommended for neonates and infants.

## 3. Unpasteurized Milk

Consumption of raw cow’s milk is not recommended nowadays because of the risk of potential pathogenic bacterial contamination. However, it is still quite popular in farming families. There are many studies that documented that unpasteurized milk may protect against allergies and asthma [17]. First observations came from cross-sectional studies conducted on Alpine farms, where school children were less likely to have asthma and atopy when they reported drinking such milk in their early childhood. These associations were independent of other protective factors connected with farm living like contact with farm animals or staying in the stables [18]. In the study of both village and town populations at all ages, raw milk exposure exerted an even stronger preventive effect on asthma and atopy in town inhabitants than among farmers and was present both in children and adults (albeit weaker) [19]. The first study trying to identify the protective biological components of raw milk was the GABRIELA survey conducted in Alpine villages on school-aged children. Consumption of unpasteurized milk in early life and currently was inversely related to asthma, but heating the milk diminished such effect. Raw milk samples taken from the farm were tested for bacterial, fat, lactose and protein content. As expected, total bacterial load was higher in raw than processed milk, but it was not associated with asthma risk, as well as total protein, fat and lactose content. Bovine serum albumin whey protein, α-lactalbumin and β-lactoglobulin were inversely related with asthma [20]. In the prospective study Protection against Allergy Study in Rural Environments (PASTURE), regular consumption of raw cow’s milk protected against asthma onset at 6 years of age. Higher-fat content milk (cream milk), butter and n-3 polyunsaturated fatty acids (PUFA) intensified this effect, especially in milder forms of asthma. According to the authors, this protective action of n-3 PUFA may be connected with their anti-inflammatory properties. Moreover, in the same cohort, a maternal diet rich in butter and unskimmed cow’s milk during pregnancy influenced the immune system of the fetus with promotion of IFN-gamma production [21].

**Figure 3:**
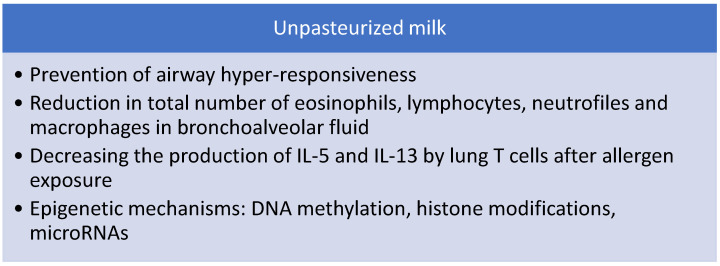
Proposed mechanism of action of unpasteurized milk in asthma primary prevention.

In an experimental model of allergen-induced asthma, raw, but not heated, milk prevented airway hyper-responsiveness and reduced the total number of eosinophils, neutrophils, macrophages and lymphocytes in bronchoalveolar lavage fluid (BAL) and IL-5 and IL-13 production by lung T cells after allergen exposure. Interestingly, both raw and heated milk were able to decrease Th2 and Th17 cells and IL-4 and IL-13 cytokines production in the lungs [22] (Figure 3). 

Some immunomodulatory cytokines present in bovine milk such as TGF-β and IL-10 may promote the production of regulatory T cells, IgA and IgG4 and inhibit IgE action. Milk proteins may also modulate microbiota composition, enhance the epithelial barrier function of the gut and influence the short-chain fatty acids production [23]. 

The components of raw milk (PUFA, bacteria, proteins and vitamins) may also act via epigenetic mechanisms. Changes in gene expression by non-DNA-encoded alterations such as DNA methylations, histone modifications and exosomes immune influence via microRNAs seem to be some of the most important anti-asthmatic and anti-allergic pathways of action [24]. Short non-coding RNA sequences (miRNAs) regulate post-transcriptional gene expression, exerting an immune regulatory function and interfering in allergic inflammation. miRNAs may potentially be implicated in the induction of the allergy lymphocytes profile and other miRNAs in promoting regulatory T cells associated with allergen tolerance [25]. They are present in raw cow’s milk and may potentially interfere with asthma genes, reducing the risk of the disease [26]. However, in the GABRIELA study, despite the differences in total miRNA quantities in farm and shop milk, it did not explain the protective effect of unpasteurized milk [27].

Bacterial endotoxins might induce tolerance towards allergens. It has been shown that the protective effect of raw milk consumption in asthma may be modified by the CD14 receptor gene polymorphism (CD14/-1721) for bacterial endotoxins. In the homozygote AA (adenine-adenine) genotype, the effect was the strongest, in heterozygous AG (adenine-guanine), it was weaker and it disappeared in children with a GG (guanine-guanine) genotype, independently of the children’s place of living [28].

The risk of severe infections after consumption of unpasteurized milk makes it impossible to conduct interventional studies comparing raw and processed milk effects on asthma prevention. However, the MARTHA trial has been recently announced, aiming to determine the protective role of minimally processed—for microbiological safety reasons—cow’s milk against standard UHT (ultra-heat treated) milk in children up to 3 years on asthma until 5 years of age [2]. The study is ongoing. We hope that this first interventional study will help in better understanding the farm milk protective effect mechanism and enable implementing practical recommendations for asthma prevention. 

## 4. Probiotics and Prebiotics

The human microbiome is one of the most important factors influencing maturation and function of the immunological system. Bacterial colonization starts in utero and may differ depending on maternal health [29]. Then, during childbirth and in the first days of life, skin, gut and lung microbial biodiversity starts to establish. This process is continued up to three years of life, then it stabilizes. Interestingly, the connection between the gut and lung microbiome, the so-called gut–lung axis, has been proposed as the possible explanation for the influence of intestinal bacteria on lung diseases [30]. The pathway of delivery (vaginal or cesarean section), environmental microbiome diversity, type of feeding, infections, antibiotic treatment or family size may have a huge influence on a child’s microbiome quality [31]. Interactions between the microbiota and the host immune system during the critical window of opportunity to develop the tolerance to potential allergens in the first year of life may exert long-lasting effects on allergy and asthma risk. It was shown that the composition of intestinal bacteria in the first weeks of life affected the risk of atopy at the age of two [32]. In the Copenhagen Prospective Studies on Asthma in Childhood2010 (COPSAC2010) birth cohort, the type of bacterial colonization of the gut at the first month of life influenced the risk of wheezing and asthma at 6 years of age. Low microbial maturity and decreased diversity of the gut microbiome had an important role in asthma risk, especially in children born to asthmatic mothers. Moreover, the mode of delivery changed the intestinal composition of bacteria in the first year of life. Higher asthma risk at 6 years of life was connected with persistent (up to one year of age) changes in the gut microbiota due to cesarean section. As the authors concluded, the composition of the gut microbiome might be changed by the delivery mode and this may have influenced the asthma risk, so the appropriate composition and maturation of intestinal bacteria seem to be important in asthma prevention [33]. 

These results suggest a potential beneficial role of specific microbial supplementation in the first year of life especially in children with higher asthma risk [34]. Probiotics and prebiotics oral application in the prenatal and postnatal periods was studied as a possible microbiome manipulation method, but the evidence is scarce. Despite some effectiveness in eczema prevention, none of the published meta-analyses could confirm the beneficial action of probiotics in reducing the risk of asthma [35,36]. Moreover, a randomized trial in a group of high-risk infants failed to prove that supplementation of Lactobacillus rhamnosus GG in the first 6 months of life prevents asthma or wheezing at 2 years of age [37]. The most recent meta-analysis found that probiotic supplementation during pregnancy or early life did not reduce the asthma or wheeze risk in infants; however, in the small subgroup of atopic infants, it significantly decreased wheeze incidence. All other factors such as type of probiotic or prebiotic used, timing of intervention, participants’ characteristics, duration of intervention and follow-up did not affect the lack of preventive effect [38].

Prebiotics are defined as “food ingredients, selectively fermented, that can cause specific changes in the composition and/or activity of the gut microbiota, thus conferring benefit(s) upon host health” [39]. Their effectiveness in reducing asthma and wheezing risk in infants was analyzed based on two randomized trials. Prebiotics decreased the risk of asthma or wheezing when compared to the control group, but because of a small number of participants and events, and inconsistencies in defining wheezing episodes and asthma, these results should be interpreted with caution [40]. Other reports did not support the protective effect of oral prebiotics in asthma [41].

All of the above observations do not support recommendation to use probiotics or prebiotics in the prevention of asthma in infants [42].

## 5. Fatty Acids

**Figure 4:**
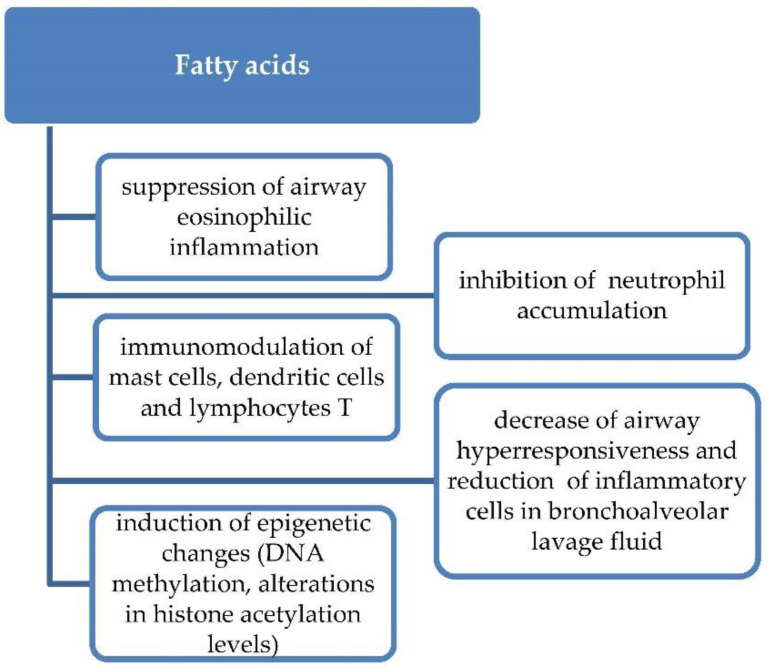
Proposed mechanism of action of fatty acids in asthma primary prevention.

Fatty acids are components of essential lipids of the human body involved in many intracellular and extracellular processes including immune modulation. Metabolites derived from omega-6 polyunsaturated fatty acids (PUFA) are associated with proinflammatory responses (leukotrienes, prostaglandins, thromboxane), while omega-3 PUFA are associated with anti-inflammatory responses (resolvins, protectins and maresins) [43]. Diets rich in fish oils, nuts, algae or flax and chia seeds are the main source of n-3 PUFA, while vegetable oils are the source of omega-6 PUFA. The Western diet is characterized by decreasing intake of n-3 long-chain polyunsaturated fatty acids (LCPUFAs) and increasing consumption of n-6 PUFA. Asthma prevalence increase took place in parallel with increasing popularity of such diet, suggesting a modifiable link between these phenomena (Figure 4).

There is an increasing body of evidence both from experimental and clinical studies that PUFA are involved in asthma inflammation at different levels [44,45]. In a murine model, inhalation of omega-3 fatty acid docosahexaenoic acid (DHA) during allergen challenge reduced the number of inflammatory cells in BAL and suppressed airway eosinophilic inflammation and airway hyper-responsiveness [46]. Moreover omega-3 PUFA-derived mediators reduced neutrophil accumulation in the inflammatory sites and directly regulated many inflammatory cells such as eosinophils, T cells, mast cells and dendritic cells. Dysregulated PUFA metabolism was observed in patients with severe asthma [47]. Recently, it was shown that n-3 LCPUFA intake in pregnancy may induce epigenetic changes in methylation levels of specific genes in offspring, regulating fatty acids desaturase and elongase, and, by that, may modulate childhood wheeze and asthma risk [48]. Moreover, maternal fish and oil consumption may influence the alteration in H3 and H4 histone acetylation levels at immune regulatory genes such as the CD14 gene in placenta or the T cell protein kinase C gene promoter in cord blood and, in this way, may regulate and modify immune responses in neonates [49,50].

In a population-based prospective cohort study, higher total and, surprisingly, n-6, but not n-3, PUFA levels during pregnancy were associated with a decreased risk of childhood asthma at the age of 6 years [51]. On the contrary, observational studies of higher maternal fish intake during pregnancy have shown beneficial effects on asthma or wheezing risk in newborns, whereas a diet deficient in n-3 PUFA increased the risk [52]. Interventional studies were undertaken to study the preventive properties of PUFAs intake during pregnancy in asthma. In a double-blind, randomized controlled study of almost 700 children, high maternal intake of fish oil capsules (rich in n-3 LCPUFA eicosapentaenoic acid (EPA) and DHA) from the twenty-fourth pregnancy week to one week after delivery significantly reduced the risk of lower respiratory tract infections, persistent wheeze and asthma in children followed up to the age of 5. This protective effect was the strongest in children of mothers with low blood levels of EPA and DHA at randomization and with the presence of a specific variant in the genes encoding fatty acid desaturase (FADS) associated with lower levels of n-3 LCPUFAs. This suggests that compensating for the deficiency may be the most beneficial after identifying such groups of pregnant women [4]. Recently, the possible underlying mechanism of n-3 LCPUFA supplementation on reduced asthma risk in this population was presented. Fish oil supplementation during pregnancy influenced the child’s metabolome by decreasing the levels of the n-6 LCPUFA pathway-related metabolites (including arachidonic acid) and metabolites of the tryptophan pathway and by increasing levels of metabolites in the tyrosine and glutamic acid pathways. These metabolic changes were associated with reduced asthma risk by age 5 and explained 24% of protective effect for asthma. Moreover, the authors studied the role of breastfeeding on the above metabolites and concluded that breastfeeding duration plays an important role in enhancing the level of n-3 LCPUFAs in children [53]. 

The long-term protective effect of maternal n-3 PUFA supplementation on asthma risk was confirmed in an interventional study with a 24-year follow-up. In this cohort, fish oil intake reduced the risk of having asthma medication prescribed or an asthma discharged diagnosis, but no association with lung function was detected [54]. On the contrary, in another randomized controlled study conducted in children with familial risk of allergies, no beneficial effect of maternal supplementation with n-3 PUFA in fish oil on any allergy and asthma outcomes was seen in a 6-year observation period [55]. 

Observational studies investigating fish intake during infancy and childhood gave conflicting results on its protective role in asthma. In some populations, high dietary intake of fish in young children decreased the risk of bronchial hyper-responsiveness and current asthma, but it was not confirmed in other studies [47]. These inconsistencies in observational surveys may be partially explained by the possibility of bias or confounding as fish consumption may represent other dietary, lifestyle or socioeconomic factors influencing the asthma risk.

In the randomized control trial of newborn infants with family history of asthma, n-3 and n-6 PUFA administration in early infancy reduced prevalence of wheeze, nocturnal coughing and bronchodilator use at 18 months, but did not prevent the onset of asthma at 5 years of age [56]. Fatty acid exposure, measured as plasma levels at 18 months, 3 and 5 years of age, dietary intake and compliance with supplements, was not associated with wheeze, atopy and current asthma at 5 years [57]. In a small, randomized study of healthy term infants, supplementation with DHA and arachidonic acid (ARA) in the first year of life reduced incidence and delayed onset of upper respiratory infection, wheezing and asthma at age 3 [58]. In a prospective study, the reduced risk of wheezing and asthma was mainly seen among 4-year-old children supplemented with n-3 and n-6 PUFA in infancy whose mothers reported a positive history of allergic diseases [59]. In another randomized study of high-risk infants, n-3 PUFA administration was associated with lower risk of recurrent wheeze, but not asthma, at 6 months of age [60]. 

The beneficial effect of fish consumption and high ratio of n-3 to n-6 PUFA on asthma prevalence reduction among young female Japanese adults was reported [61]. However, in a large Dutch adult cohort, a diet rich in n-3 fatty acids did not protect against asthma. Surprisingly, a high consumption of n-6 fatty acids significantly reduced forced expiratory volume in the first second (FEV1) of a spirometry test, most evidently in smokers. Authors concluded that the diet rich in n-6 fatty acids, rather than reduced n-3 intake, might have a beneficial effect on the lungs [62].

Short-chain fatty acids (SCFAs) are saturated aliphatic organic acids with one to six carbons, of which acetate (C2), propionate (C3) and butyrate (C4) are the most common. They are produced during fermentation of dietary fibers by the anaerobic intestinal microbiota. It is believed that SCFAs might play a role in the prevention of the so-called “diseases of civilization”. They exert anti-inflammatory effects that influence the expansion of regulatory T cells, increasing the production of IL-10 and IgA. In experimental studies, oral administration of SCFAs reduced airway hyper-responsiveness and inflammatory cells number in BAL [63]. Mice fed a high-fiber diet had increased circulating levels of SCFAs. High SCFA propionate influenced the maturation of dendritic cells in the bone marrow and inhibited Th2-mediated reactions, which reduced allergic lung inflammation upon exposure to the allergen [64]. In the PASTURE prospective birth cohort study, introduction of yoghurt in the first year of life increased the level of butyrate SCFA in fecal samples and reduced the risk of asthma in children with a high level of butyrate or propionate later in life [65]. 

Defining the ultimate role of fatty acids in asthma prevention is still ahead of us. Properly designed studies in well-defined populations are necessary to propose the practical recommendations.

## 6. Vitamins

### 6.1. Vitamin D

Vitamin D is mainly formed in the skin from 7-dehydrocholesterol, after UVB exposure. There are also foods with a high vitamin D content such as plants, fish, eggs and liver. Therefore, vitamin D is activated, firstly, in the liver and then in the kidneys. The action of vitamin D is related to its binding to VDR (vitamin D receptor) which is a ligand-activated transcription factor [66]. The receptor is located not only in bones, but also in many organs and the immune system cells. Vitamin D influences the activation of T cells, the function of antigen-presenting cells and proinflammatory cytokine production [67]. It also affects the lymphocytes Th1–Th2 balance, inhibits macrophage synthesis of IL-12 and promotes an allergic cytokine profile [68]. Vitamin D can also have a direct impact on the airways and affects their remodeling, which plays an important role in the pathophysiology and treatment of asthma [69] (Table 1).

There are some observations that vitamin D deficiency may be related to the increased incidence of asthma and allergies [70]. In a longitudinal cohort study, serum vitamin D concentrations at school age were predictive for allergy and asthma outcomes [71]. Low vitamin D levels in cord blood and in pregnant mothers increased the risk of infant respiratory infections, wheezing and asthma. Further, the lungs’ and immune system’s development in offspring was affected by vitamin D deficiency [72]. Interestingly, both low and high cord blood vitamin D levels were associated with higher risk of atopy in 5-year-old children [73].

The role of early vitamin D supplementation on asthma and wheezing development has been studied in randomized trials. In the Vitamin D Antenatal Asthma Reduction Trial (VDAART), the incidence of asthma and wheezing in children at age 3 years was lower in offspring of mothers supplemented with vitamin D in pregnancy [3]. In another cohort study, the opposite results were seen. Maternal vitamin D intake did not decrease the risk of wheezing of 3-year-old children and did not influence the asthma incidence [74]. However, in combined analysis of these two cohorts, maternal vitamin D supplementation decreased asthma and recurrent wheeze risk by 26% in children younger than 3. The reduction was stronger among mothers with higher vitamin D levels at the beginning of the study [75]. Follow-up of the VDAART cohort showed no impact of prenatal supplementation on the development of asthma and recurrent wheeze, or both, in children up to 6 years of age [76]. However, among 1- to 3-year-old children, a lower incidence of wheezing has been observed, especially in the first year of life. It has been concluded that even without a clear asthma protective effect, vitamin D intake in pregnancy may be beneficial for wheezing reduction in children with a positive family history of atopy and allergic diseases [77].

### 6.2. Vitamin A

Vitamin A takes part in many immune functions [78]. The sources of this vitamin are pre-formed vitamin A (retinol) and pro-vitamin A carotenoids [78]. In our diet, retinol comes from animal products such as whole milk, eggs and liver and also from fortified foods. The main dietary sources of carotenoids (including α-carotene and β-carotene) are carrots and orange-yellow fruits [79]. Vitamin A’s role in asthma prevention may be connected to its antioxidant potential and simultaneously to its effect on the immune system. It may regulate Th1–Th2 lymphocytes balance by reducing oxidative stress and inhibit Th17 with an antiallergic effect [80]. Vitamin A is also necessary for proper lung epithelial cell differentiation and lung development [81]. In a randomized trial, vitamin A supplementation in pregnancy and first months of life improved lung function in offspring [82]. Vitamin A deficiency could be related to a higher asthma risk. Low dietary intake of it increased the likelihood of asthma development and was associated with more severe types of the disease [83]. Vitamin A chronic deficiency early in life affected pulmonary development and promoted hyper-responsiveness, but it was not connected with an increased risk of asthma in later life. Moreover, early supplementation of vitamin A did not influence asthma risk or spirometric parameters in patients aged 9–23 years [81].

### 6.3. Vitamin C

Vitamin C (ascorbic acid) found in a variety of fruits and vegetables is used as a therapeutic agent in many diseases as it protects the immune system, reduces the severity of allergic reactions and lowers the risk of cardiovascular disease, stroke and cancer [84]. The role in asthma prevention may be a result of the vitamin C antioxidant potential and anti-inflammatory properties [79].

Regular and frequent consumption of fruits and vegetables rich in vitamin C reduced the risk of wheezing [85]. However, in a meta-analysis, even with the majority of studies documenting the beneficial effect of a fruit-rich diet on asthma, no significant protective effect of fruit intake and asthma was confirmed. Fruit intake reduced the severity of asthma in secondary prevention studies. On the other hand, vegetable intake was inversely related to asthma prevalence but not connected with the severity level. Fruit, but not vegetables, consumption was also negatively associated with risk of prevalent wheeze [86]. In a study directly comparing vitamin C blood/plasma levels between healthy and asthmatic patients, no significant difference was reported [87]. On the contrary, Harik-Khan et al. found that the risk of childhood asthma is increased by lower levels of serum vitamin C [88]. 

The final conclusions on the role of vitamin C in asthma prevention still need to be confirmed.

### 6.4. Vitamin E 

Vitamin E consists of natural isoforms and synthetic racemic isoforms, of which α-tocopherol has the highest bioavailability. Vitamin E is synthesized by plants from tyrosine and chlorophyll. People cannot synthesize or interconvert vitamin E themselves. The main sources of vitamin E are nuts, green vegetables, seeds and vegetable cooking oil. The potential anti-asthmatic effect of vitamin E may be a result of reducing oxidative stress, decreasing immunoglobulin E production and reducing airway inflammation and Th2 response by lowering IL-4 production [89]. 

Low vitamin E intake during pregnancy increased the risk of both asthma and wheezing in children for the first five years of life [90]. On the contrary, high plasma levels of vitamin E in mothers reduced asthma and wheezing risk in offspring after 2 years [91]. These results were not supported by the findings of Nwaru et al., where serum concentrations of vitamin E in the first year of life did not influence the allergy and asthma risk by age 6, except for a weak inverse association between alpha tocopherol and wheezing symptoms [92]. Studies conducted among adults also gave contradictory results, where in some, higher intake of α-tocopherol exerted a protective effect on adult-onset asthma and wheeze and improved lung function, while no such effect was seen in other populations [93]. These differences may be partially explained by the results of experimental studies in mice. The authors demonstrated opposite regulatory functions of α-tocopherol and γ-tocopherol. α-Tocopherol exerted an anti-inflammatory effect during allergic inflammation and γ-tocopherol acted as proinflammatory factor [94,95]. In a recently published cohort study, the maternal and child’s tocopherol isoform levels were measured at the second trimester and 3 years of age and spirometry tests were conducted at age 7. Higher α-tocopherol levels in 3-year-old children were related to better lung function at the age of 7 years, but only if γ-tocopherol levels were low. The protective effect of α -tocopherol vanished when γ-tocopherol levels were higher [96]. 

## 7. Selenium, Magnesium and Salt

**Figure 5:**
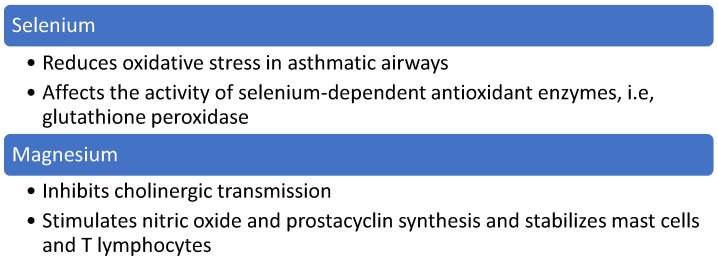
Proposed mechanism of action of magnesium and selenium in asthma primary prevention.

Other nutrients with a potential effect on asthma prevalence are selenium, magnesium and salt (Figure 5). 

Selenium has the potential to reduce oxidative stress in asthmatic airways and inflammation related to asthma pathogenesis by affecting the activity of selenium-dependent antioxidant enzymes, for example, glutathione peroxidase. This enzyme is responsible for catalyzing the reduction of hydrogen peroxide, lipid and phospholipid hydroperoxides by the antioxidant glutathione in airway epithelial lining fluid. Selenium supplementation inhibits the nuclear factor-kB activity and, in this way, limits the development of the inflammatory process in asthma [97]. In this way, a higher intake of selenium could potentially suppress asthma inflammation. Low dietary selenium intake increased asthma risk in the adult population [98]. However, the effect of selenium supplementation on objectively measured asthma symptoms was not confirmed [97]. The correlation between selenium and asthma primary prevention has not been investigated in interventional studies. 

The possible role of magnesium in the primary prevention of asthma is related to its cholinergic transmission inhibition, nitric oxide and prostacyclin, stimulation and mast cells and T lymphocytes stabilization [99]. In the current guidelines of severe exacerbations, asthma management with magnesium in a single intravenous dose is proposed to be considered in specific situations as an additional treatment. It reduced hospital admissions in patients who failed to respond to first-line treatment and with very low lung function parameters [100]. Some studies confirmed the improvement in pulmonary function in patients with severe asthma [101,102] but also magnesium action in helping to control chronic persistent asthma in children [99]. Despite this positive effect in controlling asthma exacerbations, no studies on the effect of magnesium on primary prevention of asthma were conducted and the effect is unknown. 

A low-sodium diet has been considered as a good additional therapeutic action accompanying appropriate pharmacological treatment in adults with asthma [103]. In patients with exercise-induced asthma, two weeks of low dietary intake of salt (sodium chloride) had no effect on pre-exercise pulmonary function; however, the low-sodium diet improved, while the high-sodium diet worsened, post-exercise pulmonary function values in these patients [104]. On the other hand, in a systematic review, no evidence of improving asthma control by dietary sodium reduction was shown [105]. As a low-sodium diet has many proven beneficial health effects, it is recommended regardless of its uncertain effect on asthma.

## 8. Food Allergens

Allergy to food may be a risk factor for asthma [106]. Severe or multiple food allergies increase that risk even more. Interestingly, no association between asymptomatic food sensitization and asthma prevalence was observed. Furthermore, in asymptomatic cases of atopy to food ingredients without symptoms after exposure, the asthma risk was not elevated [107]. Sensitization to eggs in infancy was a risk factor for sensitization to aeroallergens and asthma by the age of 4 years. However, it is uncertain whether an early-life allergy to eggs per se predisposes to the development of a subsequent respiratory allergy or plays a role as a marker in the course of the “allergic march” in higher-risk children [108].

In the Learning Early about Peanut Allergy (LEAP) trial, the consumption of peanuts during infancy reduced the risk of developing a peanut allergy [109]. In the same population, the effect of early peanut consumption on the development of other allergic diseases and food allergies was studied. It was found that oral tolerance induction to peanut in the LEAP study was specific for peanut allergy and early consumption of peanut had no preventive effect on development of asthma, allergic rhinoconjunctivitis or coexistent food allergies [110]. In another study of early introduction of potential allergenic food in breastfed infants, no significant benefits in food allergies protection in the third year of life were shown [111]. 

Dietary intervention along with low-level aeroallergen exposure may decrease the risk of asthma. Avoidance of allergenic foods such as milk, eggs, fish and nuts in lactating mothers and then in infants up to the age of one year simultaneously with reduction in house dust mites exposure in the environment significantly reduced allergies, asthma and eczema by the age of 1 year [112]. Moreover, such an effect was also shown at the age of 8 years in the same study population [113]. In another population, combined avoidance of food allergens, dust mites, pets’ allergens and tobacco smoke exposure reduced the risk of asthma but not allergic sensitization in children at the age of 18 months [114].

## 9. Diet Diversity and Dietary Patterns

A novel approach, considering studies on allergy prevention not only for single diet ingredients, but the whole diet model and its diversity, has been proposed recently [5]. Diet diversity means the different foods or food groups consumed in the reference period. It is believed that a more diverse diet may more effectively affect tolerance development by increasing microbial variety in the gut and by stimulation of the immunological system by different food antigens. Bacterial diversity in the intestinal flora in the first year of life was inversely associated with the risk of atopy and allergic rhinitis, but not asthma [115]. As we referred to earlier, early introduction of potential food allergens in high-risk children significantly decreased the frequency of the development of allergies [109].

In the PASTURE prospective birth cohort study, higher diet diversity in the first year of life was associated with asthma protection, decreasing the risk with each successive food added to the menu [116]. In line with these results, in another prospectively followed population from Finland, less diversity in the diet at 1 year of age increased the risk of asthma and wheeze development at age 5 [117].

Diet patterns defined as an individual’s food choice may be related to regional, ethnic, religious and cultural customs and traditions. They may also depend on socioeconomic status. Diet patterns give an overall view of food intake and may better predict the risk of diseases than individual nutrients or foods. There are different types of diets taken into consideration in the relevant studies, such as Mediterranean, Western, Japanese, seafood and vegetarian. Their effect on allergy and asthma prevention at different life stages was verified in a few surveys. A Mediterranean diet pattern (with high intake of antioxidants, n-3 PUFA, carbohydrates and fiber and low in saturated fatty acids) in pregnant mothers was not associated with the development of atopic diseases in the offspring [118,119]. However, in one study, it reduced the prevalence of persistent wheeze and atopic wheeze [120]. Studies conducted in school-aged children showed a positive effect of the Mediterranean diet on current severe asthma in girls [121] and a protective effect on asthma and wheeze [122,123]. Some studies did not confirm these observations [124]. The postulated mechanism of prevention may be related to immunomodulatory and antioxidative properties of Mediterranean diet ingredients.

The Western diet pattern in pregnancy (defined as increased intake of vegetable oil, eggs, chicken, high-salted food, processed meat and white vegetables and low consumption of fruits, soft drinks and confectioneries) was associated with a reduced risk of wheeze [125]. On the other hand, school-aged children staying on a Western diet rich in processed food and fat have a higher risk of asthma [126]. This dietary pattern was also connected with an increased risk of wheeze and asthma in other school-aged children populations [127,128]. The frequent consumption of hamburgers increased the risk of asthma symptoms and higher takeaway products intake was associated with bronchial hyper-responsiveness. These associations were independent of obesity-related factors such as BMI [129]. It has been also proposed that consumption of fast foods in childhood may decrease the protective effect of breastfeeding on asthma risk [130].

Diet diversity and particularly dietary patterns seem to play a role in asthma prevention. However, a lack of established clinical trials, inconsistencies in terminology and methodology among studies and complexity of the concept make it difficult to disentangle their final role in this process.

## 10. Conclusions and Future Perspectives

Asthma, one of the most common chronic illnesses, is a complex immunological disease with different endotype and phenotype pictures. It has a genetic background, but modifiable, environmental factors can influence its origin and course. Effective methods of asthma primary prevention are needed to control its worldwide burden and individual risk. The main strategies focus on avoiding the risk factors and promoting the beneficial exposures. Food can play both of these roles: nutrients may have a harmful effect on allergies and asthma but may also serve as a protection stimulator. That is why it is so important to understand the complex interactions between diet and immunity. Despite the efforts undertaken so far, the role of the diet in primary prevention of asthma, reflected in recommendations and practical implementations, still needs to be established.

The lack of consistency across the study results presented in this review may be due to many reasons. These are differences in study designs in terms of doses, frequency of exposure, duration, life period of intervention and quality of supplemented nutrients. The studied populations have different characteristics (genetical, environmental and sociological) and the period of observation is different. Moreover, most of these studies determined effects on asthma focusing on a single diet ingredient or food group and taking into consideration different confounders. Thus, there is a need for improvement in study methodology and design to obtain more easily comparable and interpretable data. Additional studies to define not only the role of specific diet components, but also the whole diet diversity, patterns and quality are required.

Future research should include large, multicenter, adequately powered human studies with well-defined populations, outcomes and response-influencing features (genetic, environment, immunology, microbiome, etc.). The underlying diet as a whole and nutrients interactions should also be taken into consideration. Complementary experimental studies should be undertaken to test the essential mechanisms and help to determine causal relationships in asthma protection. Such examples as promising results of epidemiological studies for unpasteurized milk in asthma prevention or results of clinical trials for vitamin D and fish oils supplementation in wheezing reduction are promising pathways for novel strategies in asthma protection, but there are still many uncertainties in the field. In the future, dietary modifications may become a new important tool to fight the asthma epidemic.

## Figures and Tables

**Table 1:** Proposed mechanism of action of vitamins in asthma primary prevention.

Vitamins	
**D**	○ Influence on activation of T cells and antigen-presenting cells ○ Inhibition of proinflammatory cytokine production and modulation of the lymphocytes Th1–Th2 balance ○ Inhibition of macrophage synthesis of IL-12 ○ Lymphocytes B suppression ○ Direct impact on the airways and remodeling inhibition
**A**	○ Antioxidant potential ○ Immunomodulation ○ Regulation of Th1–Th2 lymphocytes balance by reducing oxidative stress and Th17 inhibition ○ Influence on lung epithelial cell differentiation and lung development
**C**	○ Antioxidant potential ○ Anti-inflammatory properties
**E**	○ Reduction in oxidative stress ○ Decrease in immunoglobulin E production ○ Airway inflammation and Th2 response reduction by lowering IL-4 production

## Data Availability

Data sharing not applicable.
